# Retraction: A scientometric review of the growing trends in transcranial alternating current stimulation (tACS)

**DOI:** 10.3389/fnhum.2026.1846152

**Published:** 2026-04-09

**Authors:** Frontiers Editorial Office

The journal retracts the 6th March 2024 article cited above.

Following publication, concerns were raised regarding the scientific validity of the article. An investigation was conducted in accordance with Frontiers' policies.

It was found that the complaints were valid and that the article does not meet the standards of editorial and scientific soundness for Frontiers in Human Neuroscience; therefore, the article has been retracted.

This retraction was approved by the Chief Editors of Frontiers in Human Neuroscience and the Chief Executive Editor of Frontiers. The authors do not agree to this retraction.

